# Serum lactate as a predictor of early outcomes among trauma patients in Uganda

**DOI:** 10.1186/s12245-014-0020-9

**Published:** 2014-07-08

**Authors:** Michael Okello, Patson Makobore, Robert Wangoda, Alex Upoki, Moses Galukande

**Affiliations:** 1 Department of Human Anatomy, Makerere University College of Health Sciences, Kampala, Uganda; 2 Department of Surgery, Makerere University College of Health Sciences, Kampala, Uganda; 3 Accident and Emergency Department, Mulago National Referral Hospital, Kampala, Uganda; 4 Department of Surgery, Mulago National Referral Hospital, Kampala, Uganda

**Keywords:** Serum lactate, Injury severity, Trauma, Outcome

## Abstract

**Background:**

Trauma is the leading cause of death in the developed world. Accurate assessment of severity of injuries is critical in informing treatment choices. Current models of assessing severity of injury are not without limitations. The objective of this study therefore was to determine the diagnostic accuracy of serum lactate assays in assessing injury severity and prediction of early outcomes among trauma patients.

**Methods:**

This was a cross-sectional analytical study. Consecutive series of all eligible patients had a single venous blood sample drawn for lactate assay analysis (index test) and a concurrent Kampala Trauma Score (KTS) II value determination (reference test). Admitted patients were followed up to assess early outcomes (length of hospital stay and mortality).

**Results:**

Out of the 502 trauma patients recruited, 108 (22%) were severely injured, 394 (78%) had non-severe injuries, and 183 were admitted. There was a significant difference between median (interquartile range (IQR)) lactate levels among the severely injured (4.3 (2.6, 6.6)) and the non-severely injured (2.4 (1.6, 3.5), *p* < 0.001). After a 72-h follow-up of the admitted patients, 102 (56%) were discharged, 61 (33%) remained in the hospital, 3 (2%) remained in the ICU, and 17 (3%) had died. The area under the receiver operator characteristic (ROC) curve was 0.75 for injury severity. Serum lactate ≥2.0 mmol/l had a hazard ratio of 1.10 (*p* < 0.001) for emergency department disposition, 4.33 (*p* = 0.06) for the 72-h non-discharge disposition, and 1.19 (*p* < 0.001) for 72-h mortality. Serum lactate ≥2.0 mmol/l at admission was useful in discriminating severe from non-severe injuries with a sensitivity of 88%, specificity of 38%, PPV of 30%, and NPV of 92%.

**Conclusion:**

Hyperlactatemia in an emergency trauma patient suggests a high probability of severe injury.

## Background

Globally, injury causes 5.8 million deaths per year with more than 90% in low- and middle-income countries. In addition, trauma contributes substantially to disability and economic loss. Much of this burden could be decreased by improvements in care of the injured [[1]]. In Uganda's busiest hospital, trauma is the single most common indication of admission in the surgical wards [[2],[3]].

Accurate triage of trauma patients is a critical element of trauma systems. Current triage tools have considerate limitations and may not correlate well with severity of injury [[4]]. Available injury severity scores like the Injury Severity Score (ISS), New Injury Severity Score (NISS), Anatomic Profile (AP), Revised Trauma Score (RTS), and Kampala Trauma Score II (KTS II) base on either anatomical parameters, physiologic parameters, or both. Hyperlactatemia is not only present in severely hypotensive trauma patients but also may be seen in patients who are normotensive with multiple injuries. These patients may have severely compromised circulation and tissue hypoxia, yet because of peripheral vasoconstriction, their blood pressure may be in the normal range. Thus, for these patients, elevated lactate may serve as an early indicator of shock before blood pressure or heart rate becomes abnormal [[4]].

Despite their limitations, the Abbreviated Injury Score (AIS), ISS, and other scales still receive wide use [[5]]. Regardless of the accuracy of trauma scores, their use in clinical decision making is limited. They are complex to calculate and therefore are usually determined for purposes of audit and research. To this end, several studies have attempted to identify simpler to use (biochemical and physiological) markers that reflect physiological compromise, in order to predict morbidity and mortality [[6]–[13]].

The aim of this study therefore was to evaluate the use of serum lactate assays as an in-hospital trauma severity assessment tool and a predictor of hospital stay and mortality.

## Methods

### Study design

A cross-sectional analytical study design was used to determine the diagnostic accuracy of serum lactate assays for injury severity.

### Study setting

The study was conducted in the Emergency Department of Mulago Hospital, a large public hospital in Kampala, Uganda. The unit contains a medical and a surgical emergency wing, two operating rooms, an X-ray facility, ultrasound facility, resuscitation room with 3 beds, and a 26-bed holding emergency ward. The blood bank, hematology, microbiology, and chemistry laboratories are adjacent. On average, this emergency unit sees about 15 trauma patients daily, 12 of which have mild or moderate trauma and 3 severely injured.

### Study population

We approached for enrollment trauma patients brought to the Emergency Department who were aged 12 years and above, and presented within 24 h of injury. We excluded patients who had diabetes mellitus, cardiac disease, liver cirrhosis or malignancy, chronic renal failure, or chronic obstructive pulmonary disease. In addition, HIV-infected patients who were receiving stavudine, didanosine, or zidovudine were excluded as these drugs may elevate lactate [[14]]. Ethical approval for the study was obtained from the School of Medicine Ethics and Research Committee and Mulago Hospital Ethics and Research Committee.

Informed consent, or assent, was obtained from the patient or available next of kin.

### Study procedure

Trauma patients were managed according to ATLS protocols. The severity of injury was determined using KTS II. The KTS II scores were assigned by senior surgical residents, supervised by consultant surgeon(s). Patients requiring operative management were immediately taken to the adjacent operating theater; the remaining patients were observed for 24 h in the holding ward prior to onward transfer to the inpatient ward. Severely injured patients who needed ventilator support were initially admitted to the trauma center that has two mechanical ventilators and transferred to the ICU.

A total of 5 ml of heparinized peripheral venous blood was collected at the beginning of patient resuscitation as an intravenous access was being obtained and concurrently a sample taken for hemoglobin estimation, blood grouping and cross matching, and other laboratory tests where applicable. For lactate, a single blood sample was collected from any vein without stasis in vacuum containers with fluoride as a reagent (to inhibit glycolysis) and transported to the clinical chemistry laboratory within 15 min in a pack of ice, for analysis. Storage, if necessary, was done in a closed container at 4°C to 6°C; refrigerator was available in the adjacent blood bank.

The venous blood sample for lactate was placed on ice and transported to the laboratory within 15 min. The measurement of serum lactate was performed with a commercial kit (Randox, London, UK) on a semi-automated system 5010, using appropriate standards and quality controls. The method of determination is based on an enzymatic conversion of lactate to pyruvate and hydrogen peroxide. The hydrogen peroxide is converted to pyruvate and nascent oxygen. This nascent oxygen then oxidizes 4-amino-phenazone to a colored compound, which is measured calorimetrically. A senior laboratory technologist supervised the assay procedures.

### Statistical analysis

The investigators collected data using patient interviews, clinical findings, laboratory reports, and chart review. Demographic and clinical variables included age, sex, time of admission, mechanism of injury, time since injury, vital signs and neurological status, and KTS II score.

The predictor of interest was pre-resuscitation venous lactate level. The outcomes of interest were severity of trauma, immediate disposition at the emergency department (admitted or discharged), and 72-h disposition (discharged, still hospitalized, or dead). The Kampala Trauma Score II [[14],[15]] was used as the gold standard to stratify these patients into severely injured (KTS II ≤ 8) and non-severely injured (KTS II >8). For the purpose of this study, KTS II was dichotomized into severe (KTS II ≤ 8 includes moderate to severe injuries) and non-severe injuries (KTS II > 8 includes mild injuries).

Data were summarized using medians and interquartile ranges for continuous variables, and proportions for categorical variables. Graphical descriptive analyses were presented using box plots.

The sensitivity and specificity of lactate to stratify trauma patients according to severity of injury were estimated for various lactate level cutoffs using a 2 × 2 table and graphically presented using a receiver operator characteristic curve. We also estimated the crude and adjusted hazard ratios for early hospital outcomes using modified Cox regression models for studying hazard ratios. Lactate level was classified as 0 to 1.99, 2.0 to 3.99, and ≥4.0 mmol/l. Adjusted models included all variables that had a *p* value <0.2 in the univariate analysis. Statistical significance was defined as two-sided *p* ≤ 0.05. All analyses were performed using STATA 11.

## Results

**Figure 1:**
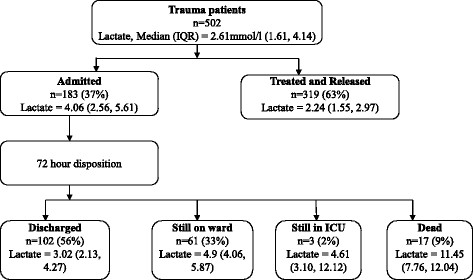
**Patient flow chart showing median (IQR) lactate levels at emergency department and 72**-**h disposition.**

We recruited 502 trauma patients for the study between February 2013 and April 2013, of which 183 patients were admitted and 319 were treated and released. The median age was 28 (interquartile range (IQR)) (see Figure 1). Seventy-nine percent of the enrolled patients were men, although the proportion of severe trauma was similar among men and women (*p* = 0.62).

At 72 h after admission, of 183 admitted patients, 102 were discharged, 61 were on the ward, 3 remained in the ICU, and 17 died.

Patients with severe trauma were more likely to be salaried workers (*p* = 0.04). The most common cause of injury was road traffic crash (269 (54%)), followed by assaults (33%). There were 443 (88%) patients with blunt trauma (see Table 1); most of the injuries were non-severe (350 (79%)).

**Table 1:** Demographic and clinical characteristics

**Characteristic**	**Kampala trauma score II**			***p*** **values**
	**Total (*n*** **= 502)**	**Severe (*n*** **= 108)**	**Non-severe (*n*** **= 394)**	
Age, median (IQR)	28 (23, 33)	28 (23, 34)	28 (23, 33)	0.530
Sex, *n* (%)				
Male	399 (79)	84 (78)	315 (80)	0.620
Female	103 (21)	24 (22)	79 (20)	
Occupation, *n* (%)				
Salaried/wage	138 (28)	21 (19)	117 (30)	0.040
Student	66 (13)	12 (11)	54 (14)	
Peasant	117 (23)	33 (31)	84 (21)	
Business	86 (17)	19 (18)	67 (17)	
Boda-boda (motorcyclist)	77 (15)	22 (20)	55 (14)	
Others	18 (4)	1 (1)	17 (4)	
Education level, *n* (%)				
None	59 (12)	23 (21)	36 (9)	0.010
Primary	214 (43)	42 (39)	172 (44)	
Secondary	146 (29)	29 (27)	117 (30)	
Tertiary	83 (17)	14 (13)	69 (18)	
Region injured, *n* (%)				
Head/neck	233 (46)	77 (33)	156 (67)	<0.001
Face	126 (25)	39 (31)	87 (69)	0.003
Thorax	53 (11)	25 (47)	28 (53)	<0.001
Abdomen/visceral pelvis	35 (7)	18 (51)	17 (49)	0.001
Extremities/bony pelvis	195 (39)	36 (18)	159 (82)	0.19
Skin	79 (16)	11 (14)	68 (86)	0.07
Cause of the trauma, *n* (%)				
Road traffic crash	269 (56)	59 (21.93)	210 (78)	<0.01
Assault	165 (33)	36 (21.82)	129 (79)	
Gunshot	3 (1)	2 (66.67)	1 (33)	
Falls	34 (7)	3 (8.82)	31 (91)	
Burns	11 (2)	6 (54.55)	5 (46)	
Others	20 (4)	2 (10.00)	18 (90)	
Type of injury, *n* (%)				
Blunt trauma	443 (88)	93 (21)	350 (79)	0.04
Penetrating	17 (4)	6 (35)	11 (65)	
Burns	10 (3)	5 (50)	5 (50)	
Others	32 (6)	4 (13)	28 (88)	
Duration, median (IQR)	3 (2, 4)	3 (2, 4)	3 (2, 4)	0.5

There was a significant difference in early outcome between the severe and non-severe groups both in terms of emergency department disposition and 72-h disposition (*p* < 0.001, see Table 2).

**Table 2:** **Outcomes at emergency department and after 72**-**h follow-up**

**Characteristic,** ***n*** **(%)**	**Kampala trauma score II**			***p*** **values**
	**Total (*n*** **= 502)**	**Severe (*n*** **= 108)**	**Non-severe (*n*** **= 394)**	
ED disposition				
Admitted	183 (36)	88 (48)	95 (52)	<0.001
Discharged	319 (64)	20 (7)	299 (94)	
72-h disposition				
Discharged	102 (56)	36 (35)	66 (65)	<0.001
Still on ward	61 (33)	33 (54)	28 (46)	<0.001
Still in ICU	3 (2)	2 (67)	1 (33)	0.11
Dead	17 (9)	17 (100)	0 (0)	<0.001

### Association between lactate levels and injury severity

**Figure 2:**
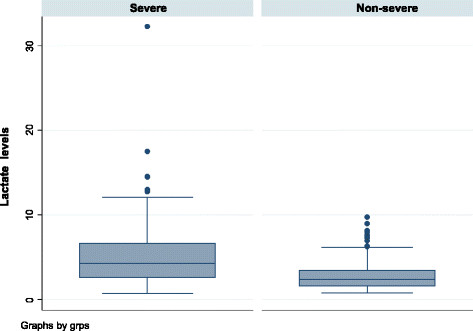
Lactate distribution between the severe and non-severe trauma patients.

**Figure 3:**
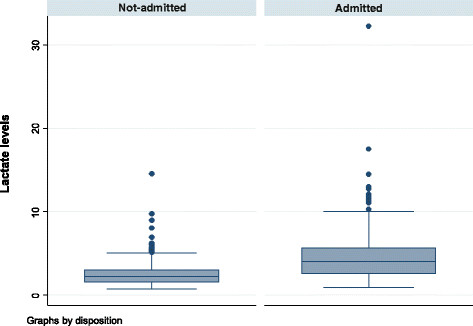
Distribution of lactate by emergency department disposition.

The median (IQR) lactate level in mmol/l was 2.6 (1.7, 4.1) among the less severely injured and 4.1 (2.6, 5.6) among those severely injured (Figure 2). Based on the lactate cutoff of 2.0 mmol/1, below that as normal and above 2.0 mmol/l as hyperlactatemia, there is a significant difference in lactate levels between the admitted and those treated and released (*p* < 0.001) (Figure 3).

### Diagnostic accuracy of admission serum lactate for injury severity as defined by the Kampala Trauma Score II

Sensitivity and specificity at different cutoffs of lactate are shown in Table 3. The cutoff of 2.0 mmol/l, at the higher end of the normal range, had a sensitivity of 88% and a specificity of 38%.

**Table 3:** Lactate level sensitivity and specificity cutoffs with corresponding positive predictive values and negative predictive values

**Cutoffs (lactate)**	**KTS II**		**Sensitivity**	**Specificity**	**PPV**	**NPV**
	**Severe**	**Non-severe**				
			0	1		
>9	18	1			94.7	80.4
			16.7	99.7		
8 to 8.99	1	3				
			17.6	98.9	82.6	81.4
7 to 7.99	6	3				
			23.1	98.2		
6 to 6.99	7	6				
			29.6	96.7	71.1	83.4
5 to 5.99	14	28				
			42.5	89.6		
4 to 4.99	18	29				
			*59.3*	*82.2*	47.8	83.4
3 to 3.99	10	58				
			68.5	67.5		
2 to 2.99	21	117				
			*88*	*37.8*	*27.9*	*92.0*
0 to 1.99	13	149				
			1	0		
	108	394				

In contrast, at a cutoff point of 4.0 mmol/l, venous lactate has low sensitivity (59%) and fairly high specificity (82%).

In the unadjusted model, lactate ≥2.0 mmol/l results in a 1.10 times (95% confidence interval (CI) 1.07 to 1.13; *p* < 0.001) higher risk of admission per unit increase in lactate. The adjusted model that also includes a lactate level ≥2.0 mmol/l has 1.75 times increased possibility of admission per unit increase in lactate at a 95% CI of 1.11 to 2.75, but the level of significance is less (*p* = 0.02). But a lactate level ≥4.0 mmol/l has 4.25 times increased possibility of admission per unit increase in lactate at a 95% CI of 2.77 to 6.52 (*p* < 0.001).

Lactate level <4.0 mmol/l was not predictive of non-discharge within 72 h, but a lactate level >4 mmol/l was, both at univariate analysis and also after adjusting for other factors at multivariate analysis; the hazard ratios were still significant (HR 31 (8 to 128)) and (HR 19 (4 to 380)), *p* < 0.001) as highlighted in Table 4 with *p* < 0.001. The initially significant characteristics like systolic and diastolic blood pressures, respiratory rate, oxygen saturation, pulse rate, primary education, and KTS II (*p* < 0.05) all became insignificant at multivariate analysis (*p* > 0.05).

**Table 4:** **Univariate and multivariate associations for 72**-**h non-discharge**

**Characteristic per unit increase**	**Univariate model**			**Multivariate model**		
	**HR**	**95%CI**	***p*** **value**	**HR**	**95%CI**	***p*** **value**
Lactate, mmol/l						
<2.0	-	Reference	-	-	Reference	-
≥2.0	4.33	0.96 to 19.51	0.06	3.70	0.81 to 16.80	0.09
*≥4.0*	*31.15*	*7.56 to 128.42*	*<0.001*	*18.53*	*4.29 to 80.00*	*<0.001*
KTS II						
>8	-	Reference	-	-	Reference	-
≤8	4.97	2.97 to 8.33	<0.001	1.56	0.73 to 3.31	0.25
Sex						
Female	-	Reference	-	-	Reference	-
Male	1.84	0.84 to 4.07	0.13	1.46	0.63 to 3.38	0.38
Occupation						
Salaried/wage	-	Reference	-	-	Reference	-
Student	0.86	0.30 to 2.44	0.78	0.84	0.29 to 2.47	0.76
Peasant	2.06	1.01 to 4.18	0.05	1.02	0.43 to 2.35	0.97
Business	1.77	0.81 to 3.88	0.15	1.03	0.45 to 2.38	0.94
Boda-boda	0.78	0.27 to 2.20	0.63	0.56	0.19 to 1.70	0.31
Others	1.24	0.28 to 5.54	0.78	0.69	0.09 to 5.27	0.72
Education level						
None	-	Reference	-	-	Reference	-
Primary	0.49	0.24 to 0.98	0.04	0.79	0.37 to 1.67	0.53
Secondary	0.46	0.22 to 0.99	0.05	0.82	0.34 to 1.98	0.66
Tertiary	0.44	0.18 to 1.08	0.07	0.85	0.28 to 2.63	0.78
Diastolic BP	0.98	0.97 to 1.00	0.009	1.01	0.99 to 1.04	0.27
Systolic BP	0.99	0.98 to 1.00	0.002	1.00	0.98 to 1.01	0.52
Per 10 unit increase						
Pulse rate	1.24	1.08 to 1.43	0.002	1.14	1.00 to 1.30	0.06
Respiratory rate	2.42	1.79 to 3.28	<0.001	1.23	0.77 to 1.98	0.38
SPO_2_	0.73	0.64 to 0.83	<0.001	0.79	0.60 to 1.04	0.09

Seventeen patients died, giving an overall mortality of 3.4% and a mortality of 9.3% among the admitted patients. Multivariate analysis was not possible due to the low numbers. However, lactate was predictive of mortality with a hazard ratio of 1.2 at 95% CI 1.14 to 1.24 (*p* < 0.001). Other significant factors were systolic blood pressure, respiratory rate, pulse rate, and oxygen saturation (see Table 4).

## Discussion

We set out to determine the accuracy of serum lactate assays in assessing severity of trauma injury and determining early outcomes in terms of length of hospital stay and mortality. We found that at a cutoff point of 2.0 mmol/l, serum lactate assays were useful in discriminating between the severely and non-severely injured trauma patients with a sensitivity of 88%. In addition it was predictive of mortality outcomes and of who would be discharged at lactate levels of 4 mmol. We used a single venous sample; the combination of single and venous as opposed to arterial and serial makes it logistically easier in low-resourced settings. Several studies have demonstrated the predictive value of a single venous sample [[16]–[18]]. We also found that the injured were mostly male with a 4:1 ratio similar to previous studies [[4],[19]–[21]] and were youthful (median age 28 years) comparable to previous studies [[22]–[24]]. Thirty-nine percent of the patients were unskilled workers (peasants and boda-boda (motorcycle) riders) similar to other studies [[19],[21],[25],[26]], and the mortality rate was 3.4%.

Blood lactate monitoring is widely used an indirect marker of tissue hypoxia for the critically ill in both emergency departments and intensive care units [[27],[28]]. Even though it is in wide use, lactate monitoring has not been consistently used across hospitals and trauma centers, a reflection of the controversy regarding usefulness of the blood sample [[6]]. Arterial, venous, or capillary samples are used in the acute setting, in addition to single or serial sampling with varying points of sampling [[16],[20],[29]].

**Figure 4:**
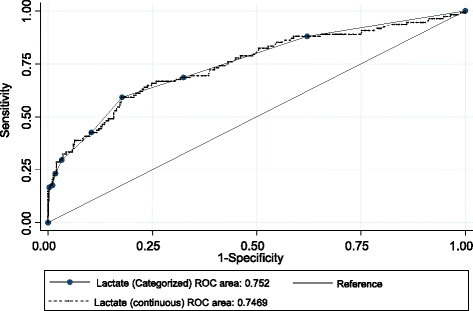
ROC curve of lactate in diagnosing severe trauma.

The area under the curve (AUC) for the receiver operator characteristic (ROC) curve for diagnosing injury severity was 0.75 in our study (Figure 4). A perfect test has an area under the curve of 1; over 0.7 is rated as good or very good for a diagnostic test [[30]].

### Predictive value of admission lactate for emergency department admission

There has been controversy over what cutoff value to use. Some researchers suggest that it should probably lie between 2 and 2.5 mmol [[6]]. Univariate analysis in our study shows that any lactate ≥2.0 mmol/l had 1.10 times increased possibility of admission per unit increase in lactate (95% CI 1.07 to 1.13, *p* < 0.001). At multivariate analysis, a lactate level ≥2.0 mmol/l had 1.75 times increased possibility of admission per unit increase in lactate (95% CI 1.11 to 2.75, *p* = 0.02). However, lactate level ≥4.0 mmol/l had 4.25 times increased possibility of admission per unit increase in lactate (95% CI 2.8 to 6.5, *p* < 0.001).

This shows a dose-response relationship between lactate level and a possibility of being admitted: the higher the lactate level, the higher the chances of admission. According to a systematic review by Kruse et al. [[6]], the dose-response relationship for single lactate measurement (pre-hospital) at the ED or in ICU with cutoff values between 2.0 and 4.0 mmol/l was seen with in-hospital mortality as primary outcome. Some studies also demonstrated a significant effect on secondary outcome measures, for instance, emergency department disposition, length of hospital stay, and the need for blood transfusion within the first 24 h. However, no independent studies are available to support or refute the relationship between admission venous lactate level and emergency department disposition.

### Predictive value of admission lactate for 72-h non-discharge from hospital

Multivariate analysis showed the possibility for non-discharge within 72 h to be 3.70 times per unit increase in lactate at a 95% confidence interval of 0.81 to 16.80. But at that level of lactate, both at univariate analysis and after adjusting for other factors, still there was no significance of lactate >2.0 mmol/l to predict 72-h non-discharge (*p* = 0.06).

At a lactate level ≥4.0 mmol/l, the possibility of non-discharge within 72 h was both significant at univariate and multivariate analyses with a 31.15 times risk per unit increase in lactate and a 95% confidence interval of 7.6 to 128.4 at univariate analysis. After adjusting for other factors like KTS II, sex, occupation, education level, systolic blood pressure, diastolic blood pressure, pulse rate, respiratory rate, and oxygen saturation, the possibility of non-discharge was 18.53 times per unit increase in lactate with a 95% confidence interval of 4.29 to 80.00, and this was significant (*p* < 0.001). At lactate level of ≥4.0 mmol/l, other initially significant factors at univariate analysis were KTS II ≤ 8, level of education being primary school, diastolic blood pressure, systolic blood pressure, pulse rate, respiratory rate, and oxygen saturation (*p* < 0.05), but at multivariate analysis, all became insignificant (*p* > 0.05).

At univariate analysis for venous lactate ≥2.0 mmol/l, Lavery et al. [[4]] found that length of hospital stay >2 days was 1.3 times more likely, but at multivariate analysis, they found it to be 0.97 times less likely. This is different from our finding where admission venous lactate was not significant at both univariate and multivariate analyses. The difference in results could be due to the fact that their outcome was length of hospital stay >2 days, yet in ours, the follow-up was up to 72 h only. Therefore, a lactate cutoff point of 2.0 mmol/l is not useful in predicting non-discharge within 72 h in trauma patients, but a lactate ≥4.0 mmol/l is useful in predicting 72-h non-discharge.

### Mortality

Out of 502 trauma patients seen, 17 (4%) died; however, the number of patients who died was not sufficient to perform multivariate analysis. At univariate analysis, however, lactate above 2.0 mmol/l had 1.19 times risk of death within 72 h per unit increase in lactate at a 95% confidence interval of 1.14 to 1.24. This was significant (*p* < 0.001). Other significant factors were systolic blood pressure, pulse rate, respiratory rate, and oxygen saturation (for all, *p* < 0.001). Thus, lactate is useful in predicting death within 72 h of admission. Lavery et al. [[4]] found that the odds of dying when lactate was ≥2.0 was 1.2 times per unit increase in lactate.

### Causes and types of injury

Road traffic crashes (RTC) were the major cause (54%), followed by assaults (33%). Odubu [[2]] found RTC to contribute 86%, Demyttenaere et al. [[22]] found RTC to contribute 61%, Renee et al. [[24]] found RTC (49%) to be the most common cause of injuries, Mutagwaba [[21]] found RTC to contribute 64% followed by assaults 25%, and Wangoda [[19]] found RTC to contribute 81%. Lavery et al. [[4]] found that vehicular injuries (51%) were the major cause of trauma in New Jersey. Locally and in the USA, RTC is still the most common cause of trauma. Blunt injuries were the majority 88%; penetrating injuries accounted for 3.4%, burns 2.99%, and others 6.4%. This is comparable to Mutagwaba [[21]] who found blunt injuries to be 88% and penetrating injuries to be 10%. Ggayi [[26]] reported blunt injuries to contribute 96%.

### Duration of injury before arrival

The median (IQR) duration was 3 h (2, 4), and this is comparable to Odubu [[2]] who found a range of 1 to 4 h, to Mutagwaba [[21]] who found a mean duration of 4 h, and to Ggayi [[26]] who found an average duration of 1.9 h in his study of SIRS in trauma patients. The duration of injury before arrival to the hospital (therapeutic vacuum) is still high in our setting as compared to other trauma centers like the Miami trauma center whose average time is 73 min. This could be because of a well-established pre-hospital care system that ensures prompt delivery of trauma patients to an appropriate trauma center after or while resuscitation is ongoing. The ambulance system is lacking in Uganda.

### Study limitations

The lack of previous studies done locally on the prevalence of hyperlactatemia among trauma patients has affected the calculation and interpretation of negative predictive values and positive predictive values which are important before drawing strong conclusions on our findings. Although the sensitivity and specificity of a test in general may have limited clinical usefulness as they cannot be used to estimate the probability of disease in an individual patient, a high sensitivity is useful for ruling in a disease if a person tests positive [[31]] and the diagnostic value of this test to the clinician will certainly be much improved if used in the context of history and clinical assessment of the injured.

Put in a different way, a test with a high sensitivity carries the risk of many false positives and hence overtriage. On the other hand, if the test is used for screening purposes or as part of a multifactorial risk assessment, it is desirable that one has a certain level of overtriage [[6]].

## Conclusion

At admission, a single venous lactate level of ≥2.0 mmol/l was useful in discriminating severely from non-severely injured patients with a fairly high sensitivity of 88%. This positive assay test would add to clinical assessment findings in determining injury severity among emergency trauma patients in resource-limited settings.

## Competing interests

The authors declare that they have no competing interests.

## Authors’ contributions

MO developed and collected the data. AU, PM, and RW supervised the research. PM and MG prepared the manuscript. All authors read and approved the final manuscript.
